# Supplementary figures and images for: Low dose of luteolin activates Nrf2 in the liver of mice at start of the active phase but not that of the inactive phase
Source: PLoS One. 2020 Apr 9;15(4):e0231403. doi: 10.1371/journal.pone.0231403 (PMC7144991; doi:10.1371/journal.pone.0231403)

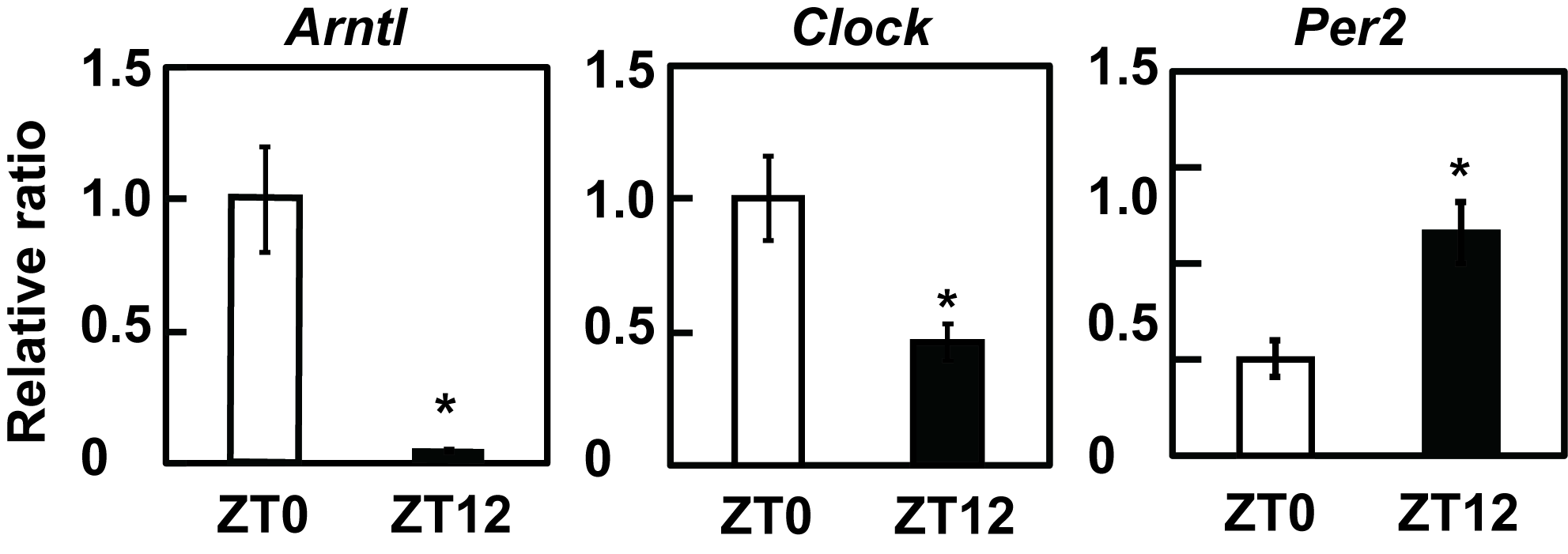

Supplement: S1 Fig — mRNA levels of Arntl, Clock and Per2 were determined by real-time PCR. The mRNA expression level was normalized by the expression of Gapdh. The results are represented as the mean ± SE (n = 6–8). Asterisks indicate a significant difference from ZT0 by the Student’s t test (p < 0.05). (TIF) [file pone.0231403.s001.tif]

Fig 1

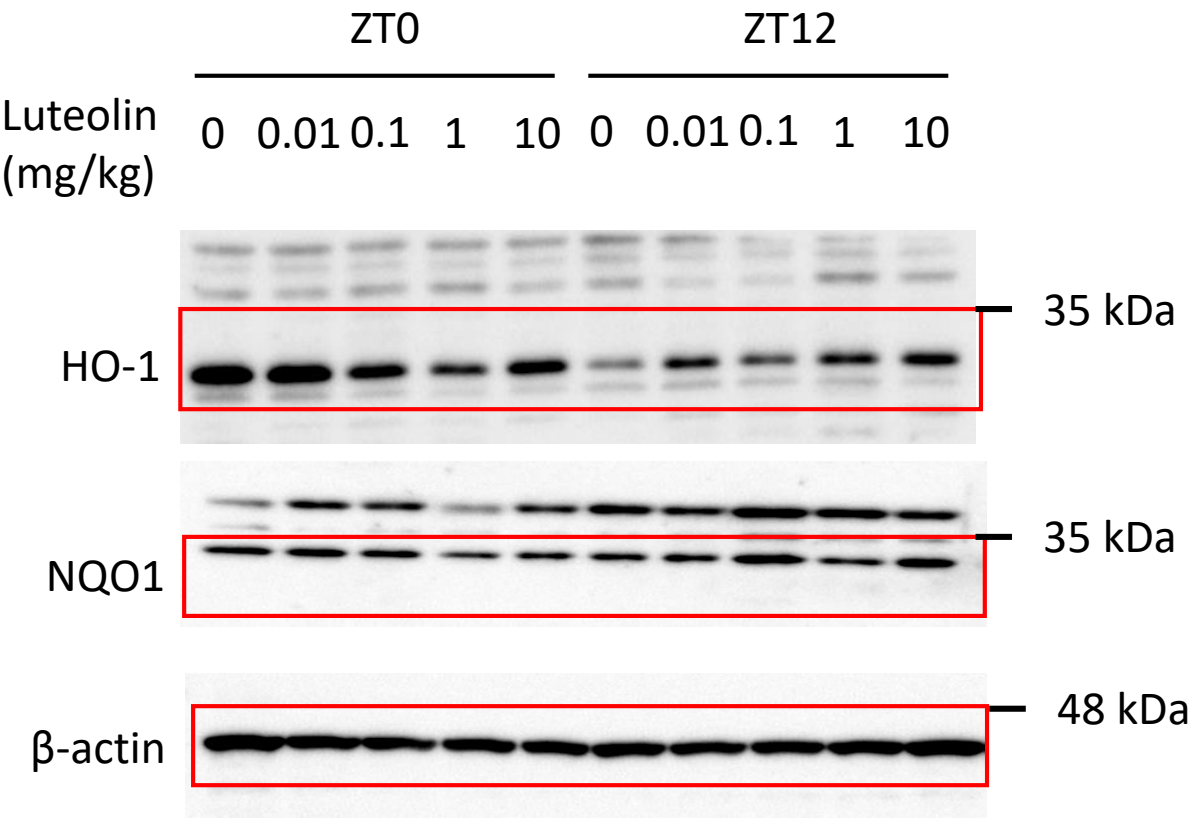

Fig 2B

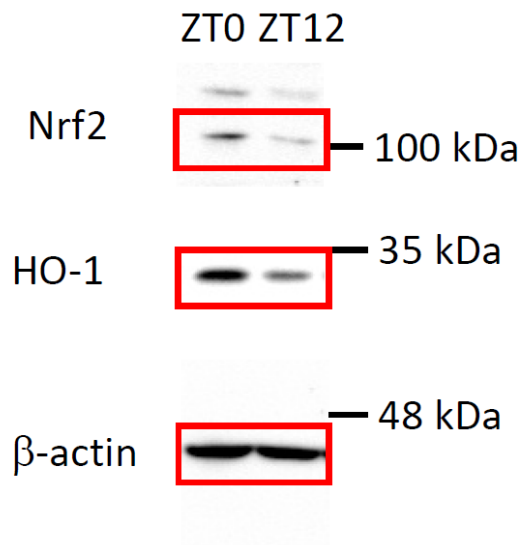

Fig 2C

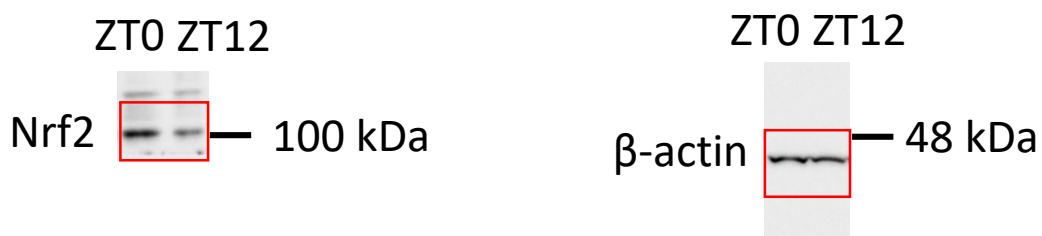

Fig 2D

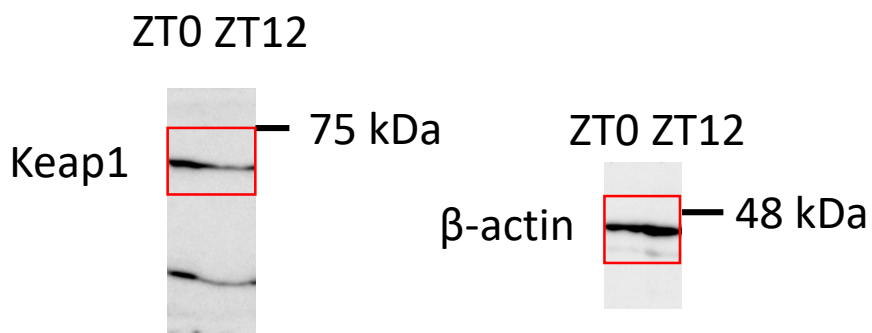

Luteolin  
(mg/kg)      0    0.01   0.1   1   10

Fig 3

Nrf2

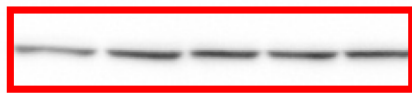

— 100 kDa

Fig 3

$\beta$ -actin

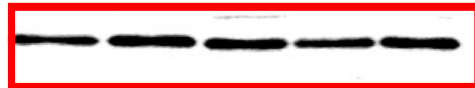

— 48 kDa

Supplement: S1 Raw Images — (PDF) [file pone.0231403.s003.pdf]
